# EU COST Action CA21130 PRESTO ‘P2X receptors as therapeutic targets’ inaugural meeting report

**DOI:** 10.1007/s11302-023-09954-x

**Published:** 2023-07-15

**Authors:** Luke Tattersall, Ankita Agrawal, Elena Adinolfi, Alison Gartland

**Affiliations:** 1 Department of Oncology and Metabolism, The Mellanby Centre for Musculoskeletal Research, The University of Sheffield, Sheffield, UK; 2 Department of Clinical Biochemistry, Copenhagen University Hospital Rigshospitalet, Glostrup, Denmark; 3 Department of Medical Sciences, Section of Experimental Medicine, University of Ferrara, Ferrara, Italy

**Keywords:** PRESTO, COST Action, CA21130, P2XR, Purinergic signalling, ATP

## Abstract

The inaugural meeting of the EU COST Action CA21130 PRESTO was held in February 2023, at the University of Ferrara, Italy. Our meeting report provides an overview of PRESTO, a tribute to Professor Jim Wiley, overviews of the talk, and a speaker synopsis that discusses the resources, models, equipment, and techniques available in different lab groups throughout Europe, increasing the prospect of collaborative research.

## Introduction

The first study recognising the physiological action of extracellular purines was published in 1929 where adenine extracts were found to regulate cardiac rhythm and blood vessel pressure [1]. However, it was not until 1972 that Professor Geoffrey Burnstock devised the term ‘Purinergic’ and showed that ATP could act as an extracellular messenger responsible for nonadrenergic, non-cholinergic transmission in the gut and bladder [2]. After the initial classification into P1 and P2, many new receptors were discovered which meant a subdivision into P2Y and P2X with the nomenclature clearly defined based on agonist potency, signal transduction, and molecular structure [3]. All seven members of the P2X receptor (P2XR) family (P2X1-7) are ATP-gated ion channels with established roles in a range of physiological and pathophysiological responses. P2XR targeting has been suggested for a variety of therapeutic applications and highly selective pharmacological agonists or antagonists, small molecule drugs, blocking antibodies, and nanobodies are available for drug discovery programs. To capitalise on the past 50 years and to harvest the accumulated knowledge in P2XR research and promote the transition to the clinic by championing the development of P2XR-targeting therapies, The European Cooperation in Science and Technology (COST) Action CA21130, PRESTO (https://www.p2xcost.eu/) has been established and is a concerted effort by leading European experts in the field of P2XRs and extracellular ATP (eATP).

Between 2022 and 2026, PRESTO ‘P2X receptors as therapeutic targets’ will support the coordination, cooperation, and knowledge exchange among basic and clinical science experts in academia and biotech/pharma industries, researchers in government/intergovernmental organisations, research councils, hospitals, and scientific communication agencies located over Europe. These joint efforts will not only lead to collaborative applications to other funding agencies, but drive the selection of the most appropriate pathologies amenable to P2XR-targeted therapy. The inaugural meeting of the PRESTO COST Action was held in February 2023, at the University of Ferrara, Italy.

This was a first opportunity for many new researchers to interact with the leading experts in the P2XR field and exchange the tools, models, and techniques available to help progress research on P2XRs towards therapy.

The meeting brought together PRESTO members from over 28 different countries with 51 speakers over 8 sessions. The talks discussed targeting of P2XRs in various aspects of human health such as in neurodegeneration and depression, inflammation and infection, cancer, and tissue regeneration, with further talks on translatability to the market and clinic. These areas constitute the four main working groups (along with dissemination of research, Working Group 5) with each working group consisting of PRESTO members overseen by two managing committee members as shown in Table 1.

**Table 1:** Working groups consisting of PRESTO members overseen by two managing committee members

Working group	Managing committee
WG1: eATP and P2XRs in inflammatory and infectious diseases	Prof Friedrich Koch-Nolte and Prof Francesco Di Virgilio
WG2: P2XRs in neurodegenerative diseases and depression	Prof Beata Sperlagh and Prof Darek Gorecki
WG3: P2XRs in cancer and tissue regeneration	Dr Valéeie Vouret-Craviar and Dr Juan José Martinez
WG4: Data integration and translation to the market and clinic	Dr Luca Antonioli and Prof Diego Dal Ben
WG5: Dissemination and outreach	Prof Alison Gartland and Dr Ankita Agrawal

The delegates were welcomed by the Action Chair Professor Elena Adinolfi, who described the structure of the Action and the various working groups. Professor Francesco Di Virgilio then paid tribute to Professor Jim Wiley who sadly passed away in December 2022.

## Professor James (Jim) Saville Wiley (9^th^ June 1938–28^th^ December 2022), The Florey Institute of Neuroscience and Mental Health, Melbourne, Australia

On December 28, 2022, our dear friend and colleague Jim passed away.

Jim was born in Sydney, and obtained his MD from Sydney University in 1973. He was Chief Hematologist at the Sydney University Nepean Hospital until 2009, when he moved to the Florey Institute in Melbourne.
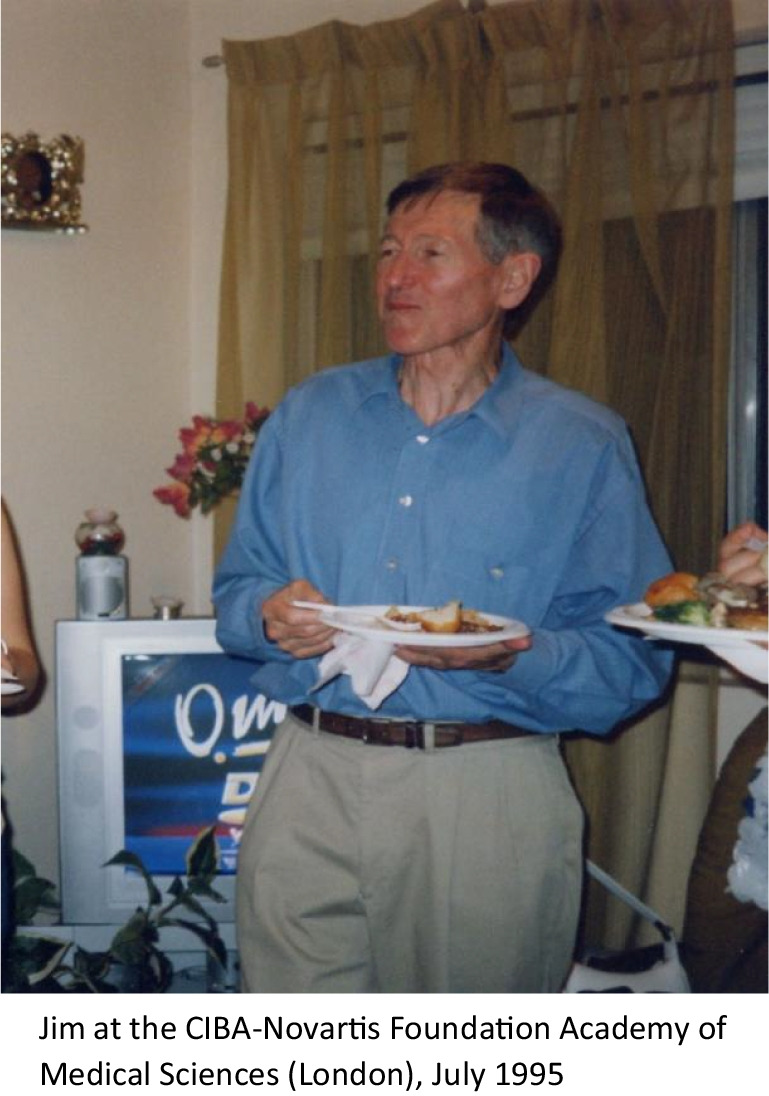


Jim was an outstanding investigator and a leading figure in the field of purinergic signalling. His initial focus of research was on the biochemistry of red blood cells, and it is interesting (and prophetic!!) that one of the first key papers he published dealt with the characterisation of erythrocyte membrane adenosine triphosphatase (Whittam R, Ager ME, Wiley JS. 1964. Control of lactate production by membrane adenosine triphosphatase activity in human erythrocytes. *Nature*, 202:1111–1112). His interest in normal and diseased erythrocyte biochemistry led him to interrogate in depth the biological significance of eATP in blood cell pathophysiology.

In 1989, he published a seminal paper with George Dubyak (Wiley JS and Dubyak GR. 1989. Extracellular adenosine triphosphate increases cation permeability of chronic lymphocytic leukemic lymphocytes. *Blood*, 73:1316–1323). This paper was the breakthrough that opened the way to all following studies leading to the identification of the role of purinergic signalling in cancer and immunity, and laid the basis for the identification of the P2X7R, at the time known as P2Z.

Ever since this early, seminal, contribution, Jim published some of the most important papers clarifying the pathophysiological function of the P2X7R. But, even more importantly, he was the initiator and the master of investigating P2X7R genetics (Gu BJ, Zhang W, Worthington RA, Sluyter R, Dao-Ung P, Petrou S, Barden JA, Wiley JS. 2001. A Glu-496 to Ala polymorphism leads to loss of function of the human P2X7 receptor. *J Biol Chem*. 276:11,135–42). We owe to Jim the identification of the most important single nucleotide polymorphisms (SNPs) in this receptor.

He further contributed to the understanding of purinergic signalling in cancer and immunity by showing how eATP promotes shedding of surface L-selectins, or how the P2X7R participates in innate phagocytosis in the absence of eATP, or in the release of circulating microparticles.

Jim’s curiosity and intellectual vivacity were not restricted to purinergic signalling. During his years at the Florey Institute, he worked on the ANZgene Consortium to genotype multiple sclerosis patients, and several prestigious publications stemmed from this collaboration.

But Jim was not just a stellar scientist. Jim was, above all, a sincere, honest, kind, generous person; a gentleman; and a friend. We all mourn his departure.

## -Francesco Di Virgilio, MD

### Overview of talks from PRESTO speakers

The first session of PRESTO began with an opening talk by Dariusz Gorecki about the role of the P2X7R in muscular dystrophy, demonstrating that blockage of the P2X7R reduced dystrophinopathy and sarcoglycanopathy in mouse models. Samuel Fountain then discussed the preclinical development and approval of gefapixant [4]; this is excitingly the first drug targeting P2XRs to be approved as a new medicine [5], gefapixant is a P2X3R antagonist used to treat chronic cough and was named to honour Professor Geoffrey Burnstock. Further talks in this session were by Gennady Yegutkin on adenosine metabolism in cancer and inflammation, Michele Zanoni on P2XRs in glioblastoma, Mehmet Uğur on intracellular signalling of the P2X7R, and Maria Domercq on the Irf5-P2X4R signalling in myelin metabolism in multiple sclerosis.

The next session opened with a talk on mitochondria and P2X7R by Mariusz Wieckowski where P2X7R intracellular localization and its effect on mitochondrial energy metabolism was discussed. Roberta Rizzo then discussed herpesvirus and host immune responses. The next two talks by Friedrich Koch-Nolte and Anna Marei Mann discussed the exciting development and applications of P2X7R nanobodies. Nanobodies are natural single-domain antibodies only made up of heavy chains, and they are naturally found in llamas and other camelids. Nanobodies have a number of useful distinctive characteristics such as a small size, high specificity, and low immunogenicity, meaning they are potentially attractive therapeutics targeting P2XRs [6]. The next talk of the session was Katarzyna Roszek discussing the role of ATP in mesenchymal and cancer cells. Other talks in this session had a particular emphasis on neurodegenerative diseases with Danijela Laketa defining the purinome in neuroinflammation, Ana Maria Sebastião discussing neuromodulation and neuroprotection by purines, and Kübra Ustaömer giving a physiatrist view of PRESTO.

The afternoon sessions again had an emphasis on neurodegenerative disorders with talks from Cécile Delarasse describing the role of P2X7R in animal models of neuroinflammation. Nadežda Nedeljković described the approaches and methodology used in her neurobiology lab group, to study purinergic signalling and ectonucleotidases in neurons, glial cells, and peripheral immune cells. Tobias Engel also described how P2X7Rs contribute to unresponsiveness to anti-seizure medications in epilepsy. In addition to these talks, Hana Zemkova discussed transmembrane domains in trafficking and dye uptake in rat P2X7R and Shai Berlin discussed how his lab group characterises mutations within NMDAR subunits and discussed the techniques, tools, and probes that could potentially be used in similar studies of P2XRs.

In the final session, two researchers from the neurobiology lab group Milorad Dragic and Marija Adzic spoke about in vivo techniques for the measurement of substance transport across the blood–brain barrier and on the use of probenecid in neuroinflammation. Continuing the trend of neurodegenerative disorders, Carlos Matute showed that P2X4R and P2X7R in microglia and/or oligodendrocytes play relevant roles in demyelination and remyelination after injury, Beata Sperlagh discussed the role of targeting P2XRs in psychiatric disorders, and Felipe Ortega the different methods to study neural stem cells lineage and purinergic signalling. Tudor Dudev also explained how lithium, which is used therapeutically to treat bipolar disorder, can act involving P2XRs. Lithium can co-bind with Mg^2+^ to ATP and form a bimetallic ATP-Mg-Li complex that can activate P2XRs with a prolonged response and may explain its therapeutic mechanism of action. Finally, one of the primary goals of PRESTO is to facilitate the progression of P2XR targeting therapies to clinical use. Clinical trials are a necessary step in the process to evaluate drug safety and efficacy. Jasmina Trojachanec Pavlovska presented an overview of the concepts surrounding design and performing of clinical trials. The end of the first PRESTO day concluded with a brief managing committee meeting.

The second day began with Juan José Martinez’s research where various in vivo and in vitro models are used to explain how activation of the NLRP3 inflammasome through P2X7R signalling confers a protection against metabolic diseases such as type II diabetes. Next, Maria João Queiroz presented the different organic synthetic methodologies that are used for the production of heterocyclic compounds with biological properties. Constantinos Athanassopoulos and Anna Pegoraro highlighted their approaches to target the metastatic potential of P2XRs with combination therapies and monitoring the vesicular cargo from tumour cells. Sahil Adriouch continued on the fascinating new application of nanobodies where data from preclinical models of colitis, experimental encephalitis, and cancer showed that AAVnano (adeno-associated viruses coding for nanobody-based biologics) targeted P2X7R for at least 120 days. The functionality, safety profile, and biological effects of AAVnano were discussed. Ankita Agrawal concluded the session by presenting how state-of-the-art tools in clinical biochemistry can be applied to propel the research on P2XR and eATP signalling.

The next session started with a focus on P2X7R where Valérie Vouret-Craviari described the activation of eATP/P2RX7/NLRP3/IL18 axis to treat pulmonary diseases, Benedetta De Ponte Conti then provided insight into P2X7R-mediated regulation of gut microbiota in cancer, which was then followed by an overview of P2X7RB variant modulation of osteosarcoma cell behaviour and metastatic properties by Luke Tattersall. A summary of ATP-P2X7R signalling in pancreatic cancer was given by Ivana Novak and Inez Zidi joined online to describe their tools to study P2X7R expression patterns in gynaecological inflammation to ultimately prevent endometrial cancer. Other talks in the session included the lysosome function of the P2X4R by Ruth Murrell-Lagnado and Joel Arrais’s take on generating novel compounds using artificial intelligence.

The first afternoon session provided an important update on the available agonists/antagonists, novel lead compounds, and the molecular modelling tools that are available within the action. The talks by Annette Nicke, Christa Müller, and Diego Dal Ben showcased the partnership of drug design and development, structural biology, and computational modelling and the establishment of biochemical and functional models to validate P2XRs as therapeutic targets in different important diseases. Mark Young highlighted the considerations of allosteric and orthosteric binding of P2X4R and P2X7R antagonists. Vanessa D’Antongiovanni and Eric Boué-Grabot highlighted the use of murine models to unravel the contribution of P2X4R in intestinal inflammation and proteinopathies affecting anxiety/memory function. This was the first introduction to the novel conditional knock-in model (P2X4KI), which could accelerate our understanding of neuronal conditions such as ischemia, chronic pain, epilepsy, multiple sclerosis, amyotrophic lateral sclerosis (ALS), or Alzheimer’s disease (AD).

The final session was kick-started by Pablo Pelegrin further highlighting the role of NLRP3 inflammasome/P2X7R in inflammation. The implication of P2XRs in skeletal myogenesis and pathogenesis of muscular dystrophies was presented by Petros Petrou; the possible therapeutic outcome of the interactions between cannabinoids and retinal neurodegenerative diseases by Relja Suručić and Victoria Maneu respectively was also discussed. Sirje Rüütel Boudinot shared their experience of validating anti-P2X4R monoclonal antibodies and Elisa Tinelli gave an overview of how they established a high-throughput screening funnel to identify CNS penetrant P2X7R blockers with a proof-of-concept study, and the lead candidate successfully rescued inflammation in their preclinical model.

## Summary

This meeting was the first opportunity for PRESTO members to meet, interact, and highlight some of the key state-of-the-art P2X research from across labs in Europe. The meeting concluded with the Action Chair giving details of the second PRESTO CA21130 COST Action meeting. This will begin with a training school for junior researchers ‘P2X receptors from basic research to market translation’ on the 4th–5th September with a second Action conference ‘P2X receptors a common route in different diseases: preclinical and clinical aspects’ from the 5th to the 7th September 2023, held in Pisa (local organiser Dr Luca Antonioli). In addition to this, it was announced that short-term scientific missions which allow scientists to conduct brief research and study visits to a research institution or laboratories in other COST countries to strengthen the existing networks and foster collaboration, learn a new technique, or use equipment, data, and/or methods not available in their own institution are now available for applications (Appendix).

## Data Availability

Not applicable.

## Appendix

Speaker synopsis on the expertise, techniques, and models that different research groups have available.

### Day 1

#### Dariusz C. Górecki School of Pharmacy and Biomedical Sciences; University of Portsmouth, UK

The research in the Górecki lab focuses on dystrophinopathy and sarcoglycanopathy, and these are the most common muscular dystrophies, causing severe disability and premature death. Acute muscle damage causes release of ATP, which activates P2X7Rs, resulting in inflammation that clears dead tissues and induces regeneration. However, in dystrophic muscles, direct or indirect loss of the α-sarcoglycan ecto-ATPase activity elevates already high eATP levels. Coinciding with P2X7R upregulation in dystrophic muscles, it exacerbates damage and hinders regeneration. Thus, P2X7R emerged as a good therapeutic target (doi:10.1186/s40478-018-0530-4). We have investigated this using hiPSc and animal models of dystrophinopathy, P2X7R knockout, and P2X7R knockin-knockout mice, with tagged N-terminus and floxed P2X7R genes (doi:10.3389/fphar.2022.935804).

#### Samuel Fountain, Biomedical Research Centre, School of Biological Sciences, University of East Anglia, UK

Our research focuses on P2XR pharmacology and physiology. We undertake high-throughput screening to identify novel ligands, using mutagenesis and molecular modelling to understand receptor-ligand interactions. Our physiological research is focused on cardiometabolic tissue and peripheral nervous system control using electrophysiology, calcium imaging, confocal microscopy, and protein chemistry in human and animal tissue to explore such roles, including P2XR KO animals and diseased patient groups. Our applied work with industrial partners involves the development of new medicines targeting P2XRs, including the recent development of the antagonist gefapixant. Through PRESTO, we will exchange knowledge and share networks to develop new tools and medicines.

#### Gennady G. Yegutkin, MediCity Research Laboratory and InFLAMES Flagship, University of Turku, Finland

Our research focuses on the role of ATP and adenosine metabolism in cancer and inflammation. We are using different approaches for studying purine-converting pathways in vitro and ex vivo, including thin-layer chromatographic assays with radiolabelled nucleotides and adenosine, enzyme-coupled purine-sensing assays, and in situ enzyme histochemistry, as well as phenotypic identity and spatial distribution of key ecto-nucleotidases using high-resolution 3D multiplexed imaging. By combining these methods with signal transduction assays conducted by other PRESTO members, we hope to develop novel therapeutic strategies targeting nucleotide-inactivating ectoenzymes and P2XR as key components of the disordered ATP metabolic and signalling network in cancer.

#### Michele Zanoni, Translational Oncology Unit, Biosciences Laboratory, IRCCS Istituto Romagnolo per lo Studio dei Tumori (IRST) ‘Dino Amadori’, Meldola, Italy

Our research approach is both preclinical and translational. Thanks to our strict collaboration with clinicians, we have access to biological samples from patients and clinical data. Our aim is to identify circulating and tissue biomarkers related to prognosis and response to therapies, using tumour tissue, liquid biopsy, and next-generation sequencing technologies. In addition, we use in vitro (organoids, spheroids, and primary cell cultures) and in vivo models (zebrafish and mice) to study mechanisms of tumour progression, resistance to therapies and metastatic dissemination. We have studied the role of P2X7R splice variants in relation to radiation therapy resistance in glioblastoma. During PRESTO, we hope to share skills and knowledge with the other members to establish new collaborations and advance research on P2XRs in the oncology field.

#### Mehmet Uğur, Department of Biophysics, School of Medicine, Ankara University, Ankara, Turkey

Our research focuses on the intracellular signalling mechanisms of P2X7R. Contrary to the established belief that P2X7R is a non-selective cation channel and its signalling action is through flux of small cations like Ca, new data suggests that this receptor may additionally be interacting with other intracellular pathways in a more direct way to activate some cellular responses. One such candidate is a P2X7R-activated anionic transporter in macrophages. If this suggestion is proven, this will open up the possibility to development of biased ligands for this receptor. We hope that PRESTO interactions will help develop this idea.

#### Maria Domercq, University of the Basque Country, Leioa-Vizcaya, Spain

Our research focuses on the role of the purinergic P2X4R in multiple sclerosis. We use transgenic mice in which we manipulate surface P2X4R expression, provided by Eric Boué-Grabot (University of Bordeaux). We have studied the role of P2X4R and its signalling pathways in different microglia function such as motility, chemotaxis, and phagocytosis. We have also developed and characterised using electrophysiology new allosteric modulators of P2X4Rs, with the potential to advance to preclinical studies. During PRESTO, we hope to exchange both knowledge and technology to advance the research on P2X4Rs in multiple sclerosis and other pathologies.

#### Mariusz R. Wieckowski; Laboratory of Mitochondrial Biology and Metabolism, Nencki Institute of Experimental Biology, Polish Academy of Sciences, Warsaw, Poland.

Our research focuses on mitochondrial metabolism, neurological disorders, and non-alcoholic fatty liver disease (NAFLD). We investigate intracellular localisation of the P2X7R especially focusing on mitochondria and its effect on mitochondrial energy metabolism. We are looking for possible alterations in the level of P2X7R in the neurodegeneration with brain iron accumulation using NBIA patients’ fibroblast. Additionally, in rodent models, we are investigating the involvement of the P2X7R/inflammasome NLRP3 axis in the NAFLD progression. We perform complex characterisation of mitochondrial respiratory chain function and the related parameters responsible for or involved in mitochondrial defect-mediated cellular dysfunction.

#### Roberta Rizzo, Department of Chemical, Pharmaceutical and Agricultural Sciences, University of Ferrara, Ferrara (Italy)

Our research focuses on virology, immunology, molecular biology, and neuroimmunology, with high expertise in herpesvirus impact on host immune system. We have different 3D organoid models (e.g. brain, lung). We have access to the Laboratory for Advanced Therapy Technologies (http://ltta.tecnopoloferrara.it/), which makes use of a structure dedicated to experimentation in animal models (LARP) (surgical, behavioural, in vivo imaging analyses, cellular and histological analysis, etc.) and in the BSL3 laboratory structured for both in vitro and in vivo studies. During PRESTO, we hope to exchange our expertise and interests with other collaborators.

#### Anna Marei Mann, Institute of Immunology, University Medical Center Hamburg-Eppendorf (UKE), Hamburg, Germany

Our research aims to improve the cell specificity of AAVs by using nanobodies, the single antigen binding domain derived from camelid heavy-chain antibodies. We genetically fused P2X7R-specific nanobodies into a surface exposed loop of the VP1 capsid protein, resulting in the display of up to five nanobodies on the AAV capsid. We also generated bispecific adaptors consisting of a P2X7R-specific nanobody fused to an AAV-specific nanobody. These nanobody-displaying AAV and nanobody-based adaptors markedly enhance the transduction of P2X7R-expressing cells. During PRESTO, we hope to exchange knowledge and technology to improve targeting and functional modulation of P2X7R-expressing cells.

#### Danijela Laketa, Institute for Physiology and Biochemistry, Faculty of Biology, University of Belgrade, Belgrade, Serbia

We study the role of ectonucleotidases CD39, CD73, and NTPDase2, as major regulators of eATP, eADP, and eAdo in the CNS, together with P2 and P1 receptors in neuroinflammation, in the acute EAE model. We also use microglial and oligodendroglial cell lines and primary astrocyte cultures. The techniques we utilise are real-time PCR, western blot, enzyme assays, immunohisto- and immunocytochemistry, in situ hybridization, and fluorescent and light microscopy. We hope PRESTO will increase the exchange of expertise and improve the skills among members, boosting the successful translation of laboratory findings on P2XRs, into the clinical development of new biomarkers and therapeutics.

#### Katarzyna Roszek, Department of Biochemistry, Faculty of Biological and Veterinary Sciences, Nicolaus Copernicus University in Torun, Poland

Our research is focused on the purinergic context of autocrine/paracrine signalling in mesenchymal stem cells (MSCs), cancer cells, and in the cross-talk between them. We also postulate a critical role of ecto-nucleotidases and ecto-kinases which orchestrate a fine-tune regulation of nucleotide signals. We have human normal (fibroblasts, MSCs) and cancer cell lines (e.g. glioblastoma, lung, breast, and colon) available for in vitro culture. We can analyse cell viability, senescence, migration, nucleotidases, kinase activities and gene and protein expression. We hope to share the experience of PRESTO members and contribute to the understanding of the purinergic signalling dynamics involving eATP and P2X7R.

#### Kübra Ustaömer, Tekirdağ Namık Kemal University Hospital, Department of Physical and Rehabilitation Medicine, Tekirdag Namik Kemal University, Turkey

As a medical specialty, physical and rehabilitation medicine is responsible for the prevention, diagnosis, treatment, and rehabilitation management of people with disabling medical conditions and comorbidity across all ages. Dealing with many painful conditions and pain syndromes, inflammatory conditions, regeneration processes, and medical, physical, and exercise treatment options in various diseases leads us to follow innovations and developments in these fields. Of particular interest are the effects of purinergic signalling on exercise, inflammation, and pain. During PRESTO, we hope to utilise the skills and expertise of the members and collaborate on clinical research on P2XRs and chronic pain, inflammation, and exercise.

#### Ana Sebastião, Institute of Pharmacology and Neurosciences, University of Lisbon, Portugal

Our team aims to elucidate how the neuronal and glial components of the tripartite synapse are fine-tuned under normal and dysfunctional situations. How endogenous modulators affect synaptic signalling, neuronal excitability, neuronal and glial maturation, degeneration, renewal, and repair. We focus on adenosine and its interactions with neurotrophic factors and endocannabinoids. For disease models, we have been interested in those involving neurodegeneration, neuroinflammation, and/or dysregulated excitability, namely epilepsy (including absence epilepsy), Rett syndrome, amyotrophic lateral sclerosis, Alzheimer’s disease, and multiple sclerosis. During PRESTO, we aim to interact with experts about P2XRs, to understand its contribution to some of these diseases.

#### Cécile Delarasse, Institut de la Vision, Sorbonne University/Inserm, Paris, France

Our research focuses on immunological and inflammatory processes in retinal diseases and the role of P2X7R in different models of neurological diseases. We have various available in vivo models of eye diseases and conditional and cell-specific P2X7R-deficient mice. We are equipped with many platforms to phenotype cells and tissues: flow cytometry (analysis and cell sorting), RNA sequencing, and single-cell RNAseq. PRESTO will allow us to share our knowledge and our technical skills in order to develop innovative therapeutic approaches to target P2XRs.

#### Hana Zemkova, Department of Pain Research, Institute of Physiology of the Czech Academy of Sciences, Czech Republic

Our laboratory focuses on research of membrane receptors and ion channels in pituitary cells and neurons. We use electrophysiology, microfluorimetric methods, transfected HEK-293 T cells, rat brain slices, and inflammatory or neuropathic pain models. During PRESTO, we will focus on identification of new neurosteroids that display modulatory, and possibly also receptor-specific inhibitory effects on P2XRs. We have a large library of P2X2R, P2X3R, P2X4R, and P2X7R mutants that might be useful for localisation of binding sites that we can offer to collaborating PRESTO members. Characterisation of binding sites for new molecules might be important for the design of new drugs in future.

#### Tobias Engel, Department of Physiology and Medical Physics, Royal College of Surgeons in Ireland, Dublin, Ireland

The main focus of my research group is the study of purinergic signalling during seizures and epilepsy with a particular focus on the ATP-gated P2X7R. Using a combination of pre-clinical in vivo mouse models and patient material (brain tissue and blood samples), we have shown that blocking of the P2X7R not only reduces epileptic seizures and underlying neuroinflammation, but also contributes to unresponsiveness to anti-seizure medications. The ongoing work of my research is now trying to understand how P2X7R contributes to seizures via the use of cell type-specific P2X7R knockout mice and electrophysiological approaches in patient tissue. In addition to treatment, we are also investigating the diagnostic potential of purinergic signalling as a tool for epilepsy, which includes P2X7R-based positron emission tomography (PET) imaging and the analysis of blood purine concentration changes. PRESTO offers us the unique possibility to expand our analysis of purinergic signalling during epilepsy via the use of new methods to modulate P2X7R activation during disease progression and to analyse P2X7R down-stream signalling during seizures, critical to advance P2X7R-based therapies towards a clinical application.

#### Nadezda Nedeljkovic, Laboratory of Neurobiology, Department of General Physiology and Biophysics, Faculty of Biology University of Belgrade (https://brainpurines.bio.bg.ac.rs).

Our group studies how purinergic signalling mediates interactions between neurons, glial cells, and peripheral immune cells during neuroinflammation. We study the role of CD39, CD73, P1, P2X, and P2Y, and their complex regulation at the neurovascular unit. We use several in vitro models (primary astrocytes, BV2, and OliNeu cell lines) and animal models of acute and chronic neuroinflammation (EAE, 6-ONDA, and TMT). We use stereotactic surgery in conjunction with cerebral open-flow microdialysis, tissue isolation, purification, subcellular fractionation, qRT-PCR, WB, functional assays, immunofluorescence, and confocal microscopy. P2X4R and P2X7R will be the focus of our upcoming research collaborations within PRESTO.

#### Shai Berlin, Department of Neuroscience, Ruth and Bruce Rappaport Faculty of Medicine, Technion—Israel Institute of Technology, Israel

The Berlin lab at the Faculty of Medicine of the Technion is a molecular neuroscience lab, with a strong focus on the development of genetic and chemical tools to study the brain at various scales, in both health and disease. We study how synaptic receptors affect the function of the synapse and explore how patient-derived mutations within synaptic receptors instigate diseases. We are particularly interested in NMDARs, how mutations affect receptor function and expression, and the roles of synaptic vs. extrasynaptic receptors. These studies are complemented by the development of unique molecular, chemical, and genetic ‘tools’ to examine synaptic and neural function in vitro and in vivo. Our work lies at the intersection of chemistry, biology, and medicine. For in vitro studies, we produce primary neuronal cultures from rats, mice, and quails and extract oocytes from *Xenopus Laevis* frogs for two-electrode voltage clamp (TEVC) electrophysiology. For in vivo studies, we employ mice, zebrafish, and quails. At our immediate disposal are methods such as electrophysiology (TEVC and patch-clamp electrophysiology); confocal-, light sheet-, and a two-photon microscope; molecular biology; and AAV production. Our faculty provides us with MRI, micro-CT, CyTOF, mRNA sequencing services, super-resolution, and EM microscopy. Through the PRESTO network, we will expand our studies towards novel receptor targets by establishing strong collaborations, student exchange programs, meetings, etc. Aside knowhow, we will certainly gain colleagues and friends. We intend to apply our expertise and knowhow to explore P2XRs and their implication in diseases (especially of the brain, such as gliomas), and to help explore novel therapeutic approaches and delivery methods (such as AAVs).

#### Carlos Matute, Laboratory of Neurobiology, Achucarro Basque Center for Neuroscience, CIBERNED and University of País Vasco (UPV/EHU), 48,940-Leioa, Spain

We study the molecular and cellular mechanisms of neurodegeneration. Our in vitro models include murine and human cell cultures of neural cells alone or in combination, organotypic and acute slices, and human cerebral organoids from controls and patients. Our in vivo models include experimental multiple sclerosis (autoimmune encephalitis, cuprizone, and lysolecithin), stroke (MCAO), and Alzheimer’s disease (3xTg-AD). The techniques at our disposal comprise immunochemistry, mice and human histology at light and electron microscopy levels, mitochondrial metabolism, oxidative stress, calcium imaging (cultures, slices, 2-photon microscopy), and functional assays (electrophysiology). The P2XRs I will focus on during PRESTO are P2X4R and P2X7R particularly in multiple sclerosis. We found that these receptors acting at diverse cellular compartments could mediate oligodendrocyte and myelin damage and promote tissue repair. PRESTO offers us a unique opportunity to learn about the role of these receptors in oligodendrocyte and myelin biology, and to deepen those findings in multiple sclerosis with the ultimate goal of bringing them to the clinic with newly developed drugs targeting P2X4R and P2X7R. I plan to interact with drug developers to find out about novel ligands (agonists, antagonists, positive allosteric modulators) acting at P2X4R and P2X7R that are ready for functional testing both in vitro and in vivo. I open my lab to young and senior investigators to share knowledge, techniques, and equipment. I look forward to establishing solid collaborations that can compete for substantial international funds.

#### Todor Dudev, Faculty of Chemistry and Pharmacy, Sofia University ‘St. Kliment Ohridski’ 1164 Sofia, Bulgaria

Our expertise is in the field of molecular modelling of structures/processes of interest to chemistry, biology, and medicine. Various theoretical approaches have been employed. Special attention is paid to studying the therapeutic activity of abiogenic metal cations (Li(I), Ag(I), Sr(II), Ga(III)) in fighting psychiatric disorders, tumour formation, and bacterial infections. Another line of research is focused on studying the thermodynamics/kinetics of bioactive molecules entrapped in macrocyclic hosts. In the current PRESTO action, we could contribute with modelling some processes related to P2XR activity, as well as those connected to drug delivery mechanisms (by cage molecules) of promising therapeutics against diseases, involving P2XRs.

#### Felipe Ortega and Rosa Gómez Villafuertes, Department of Biochemistry and Molecular Biology, Faculty of Veterinary Medicine, Universidad Complutense of Madrid, Instituto Universitario de Investigación en Neuroquímica (IUIN) and Instituto de Investigación Sanitaria San Carlos (IdISSC) Madrid, Spain.

Our group studies the mechanisms driving the generation of the neural progeny. We aim to elucidate which intrinsic and extrinsic factors are responsible for modulating this process and their relation to the onset of pathological scenarios (Developmental Cortical malformations, neurodegenerative diseases). Accordingly, we have developed methods that we aim to provide to fruitful collaborations within the PRESTO network. These methods combine the culture of NSCs with continuous live imaging by time lapse video microscopy to study embryonic, postnatal, and adult neurogenesis on both murine and human models, focusing on purinergic signalling (specially P2X7R) and its associated modulating and therapeutic potential.

#### Marija Adzic Bukvic, Laboratory for Neurobiology, Faculty of Biology, University of Belgrade, Serbia

Our research group focuses on the role of purinergic signalling in glia-derived neuroinflammation and how different pharmacological agents targeting ectonucleotidases and P2X and P1 receptors can modulate it. We use in vitro models of glia-derived neuroinflammation on primary cultures of astrocytes and microglia, scratch wound assays as a model for mechanically induced injury, and on glioblastoma cell lines. We routinely use immunoblot, immunocytochemistry, biochemical enzyme assays, calcium imaging, and microscopy. We hope that by sharing expertise among members of PRESTO, we can form collaborations to elucidate the role of the P2X7R and how its blockade (i.e. probenecid) may modulate glia-derived neuroinflammation, as well as the mechanisms responsible for it.

#### Jasmina Trojachanec, Department of Preclinical and Clinical Pharmacology and Toxicology, Medical Faculty, Skopje, R.N. Macedonia

Our research focuses on preclinical experimental research on various animal models (rats and mice), at the molecular and receptor level and clinical studies in healthy volunteers and patients. Safety, followed by efficacy in clinical trials, has been in our primary focus. We saw an opportunity to be actively involved in the development of research related to new therapeutic possibilities targeting P2XRs for patient benefit. We hope that through sharing skills and expertise among PRESTO members that we can form collaborations that will improve translatability of targeting P2XRs for patient benefit.

#### Milorad Dragic, Laboratory for Neurobiology, Faculty of Biology, University of Belgrade, Serbia

Our research group focuses on the role of purinergic signalling in neuroinflammation and how it contributes to the transition from acute to chronic states in various neurodegenerative diseases. We routinely use in vivo models of neurodegeneration such as the 6-hydroxydopamine-induced model of Parkinson’s disease, the trimethyltin-induced model of Alzheimer’s disease, experimental autoimmune encephalomyelitis as a model for multiple sclerosis, and ischemic reperfusion as a model for transient ischemic attacks. We routinely use stereotactic surgery, immunoblot, immunohistochemistry, biochemical enzyme assays, and cerebral open-flow microdialysis. We hope that by sharing expertise among PRESTO members, we can form collaborations to elucidate the role of the P2X7R in acute and chronic neuroinflammatory conditions that accompany all neurodegeneration.

### Day 2

#### Juan Jose Martinez-Garcia, Department of Biochemistry and Molecular Biology B and Immunology, University of Murcia, Biomedical Research Institute of Murcia, Murcia, Spain.

Our research focuses on innate immunity and the inflammatory response promoted on several autoimmune, neurodegenerative, infectious, and metabolic diseases. Currently, we have well established the in vitro inflammasome activation in monocytes and macrophages as a two-signal response system where P2X7R is activated as a second signal after TLR4 signalling. Additionally, we have at our disposal deficient mice in NLRP3 inflammasome and Gasdermin D. Both in vivo and in vitro models can be explored by different techniques from cell culture and flow cytometry, molecular biology, protein detection, microscopy, etc. During PRESTO, we aim to exchange our knowledge and tools to establish fruitful collaborations to get a better understanding of P2X7R as a possible therapeutic target for inflammatory diseases.

#### Maria-João Queiroz. Centro de Química Universidade do Minho, Portugal

The expertise of my group is in organic synthesis, namely the synthesis of fused heterocycles of nitrogen and/or sulphur, with different functionalisations. We are able to synthesise new molecules preceded by in silico studies for interactions with P2XRs. After the appropriate studies either in vitro or in vivo, we aim at contributing to potential therapies that involve the referred receptors. We have already obtained good results in the synthesis and evaluation of new potential anti-tumour and anti-angiogenic compounds, working in collaboration with computational and biological research groups having the tyrosine kinase domain of VEGFR2 as a target.

#### Anna Pegoraro, Department of Medical Science, University of Ferrara, Italy

Our research focuses on P2X7R and the other members of the purinergic-adenosinergic axis in oncogenesis. We have investigated P2X7R-dependent vesicle release from cancer cells and evaluated their miRNA content and effect on proliferation and migration by scratch and wound healing assays. We study the effects of P2X7R antagonists in models of primary and metastatic solid and liquid tumours, focusing on immune infiltrate and live eATP measure in the tumour microenvironment. Through collaborations established from the PRESTO network, we hope to identify neoplasias amenable to P2XR-targeted therapies and provide preclinical evidence for P2XR-centered clinical trials.

#### Sahil Adriouch, Faculty of Health, Department of Immunology and Biotherapy, University of Rouen Normandie, Rouen, France

We have recently developed a methodological approach (termed AAVnano) based on AAV vectors coding for selected nanobody-based biologics with the aim to rapidly validate their functionality, their safety profile, and their long-term biological effects directly in vivo. The AAVnano approach has been applied to appreciate the importance of P2X7R as a target in acute colitis, in experimental autoimmune encephalomyelitis (EAE), and in different tumour models. In collaboration with PRESTO members, we aim to further validate the importance of P2X7R and other P2XRs as druggable targets in various diseases, and to extend this approach to other key purinergic players (e.g. CD73, CD39) against which specific nanobodies have recently been generated.

#### Ankita Agrawal, Department of Clinical Biochemistry, Capital Region, Denmark

Our research approach is both analytical and interpretative. We are equipped with numerous platforms to examine components in body fluids/tissues and put them in the context of a disease, i.e. diagnosis, screening for susceptibility, monitoring treatment progress, thus, covering various research areas. In the wet lab, using cell culture and rodent models, we aim to understand the purinergic signalling dynamics involving ATP and P2X7R causing drug resistance and relapse in patients with a type of blood cancer that attacks bones. During PRESTO, we hope to exchange both knowledge and technology to advance the research on P2XRs towards therapy.

#### Valérie Vouret-Craviari, Institute of Research on Cancer and Aging, University Cote D’Azur, France

Our research focuses on lung diseases mostly lung cancer and fibrosis. We have lung cancer cell lines (NSCLC and SCLC) from murine and human origin and immunocompetent lung tumour mouse models. We also have a chemical drug able to enhance the activity of P2X7R both in vitro and in vivo, and we recently described a procedure allowing the measurement of P2X7R in vivo. We showed that the P2X7R/NLRP3/IL18 pathway favours anti-tumour and anti-fibrotic immune responses in mouse preclinical models. During PRESTO, we hope to exchange both knowledge and technology to advance the research on P2X7Rs in lung diseases.

#### Benedetta De Ponte Conti, Mucosal Immunology lab, Institute for Research in Biomedicine, Bellinzona, Switzerland

Our research line focuses on understanding the role of eATP and purinergic signalling in the gut, and how they shape immune response and bacterial composition in different pathological conditions. To address this, our lab exploits in vivo models of intestinal dysbiosis, cancer immunotherapy, and immunisation against enteropathogens combined with the oral delivery of an *E. coli* engineered to release the ATP-diphosphohydrolase (apyrase), which is able to catalyse the conversion of ATP into AMP and to reduce concentrations of eATP in the gut. During PRESTO meetings, we hope to create collaborations and expand our knowledge of eATP and purinergic signalling for patient benefit.

#### Luke Tattersall, The Mellanby Centre for Musculoskeletal Research, Department of Oncology and Metabolism, The University of Sheffield, UK

Our research focuses on bone physiology, cancer, and metastasis. We have various bone, breast, and prostate cancer cell lines available for both in vitro and in vivo models and can further establish PDX models of bone cancer. We have studied the role of P2X7R and its splice variants using a number of bone and cancer assays such as mineralisation, ALP activity, migration and invasion, micro-CT, and histology/IHC. We hope that through sharing skills and expertise among PRESTO members that we can form collaborations that will improve translatability of targeting P2XRs for patient benefit.

#### Ruth Murrell-Lagnado, University of Sussex, Brighton, UK

Our research focus is on the effect of purinergic receptors on lysosome trafficking. P2X4R acts as a Ca^2+^ permeable channel at the lysosome membrane in addition to the plasma membrane and in doing so plays a role in regulation of lysosome trafficking and function. We have studied the regulation of these lysosomal channels and their contribution to the adaptation of the lysosomal system in breast cancer cells, using targeted calcium-reporter proteins and live cell fluorescence imaging. Lysosomal P2X4R activity is triggered downstream of P2X7R activation and promotes Ca^2+^ release from lysosomes. In breast cancer cells, expression of P2X4R affects lysosome membrane trafficking, cell proliferation, and invasiveness. Hence, this receptor is an important therapeutic target.

#### Inès ZIDI, Laboratory of Microorganismes and Active Biomolecules, Sciences Faculty of Tunis, University of Tunis El Manar, Tunis, Tunisia

Our research approach is directly related to the analysis of patient tissue and blood. We work on digestive and gynaecological cancers collected from Tunisian Patients. Techniques at my disposal include proteomic techniques (i.e. immunohistochemistry, ELISA) and molecular and next-generation molecular techniques. During the PRESTO project, we will focus on P2X4R, P2X5R, and P2X7R expression in different subgroups of patients (after stratifications by clinical pathological features) and analyse their relationship with the tumour microenvironment. We will analyse the interaction of P2XRs with the expression of non-classical HLA and other immune tolerance molecules. Finally, we will look for liquid signatures associated with changes in P2XR expression. All analysed features will be checked for their potential in cancer diagnosis and prognosis.

#### Mark Young Cardiff, University, Cardiff, UK

Our research focuses on using structure-based design to develop novel small molecule modulators of P2X7Rs, and understand how these modulators bind to influence receptor function. To address this, we use a combination of molecular modelling, computational docking, mutagenesis, and functional assays in transfected mammalian cells. We are also interested in the role of P2X7Rs in the progression of inflammatory eye disease and are developing ex vivo models to study patterns of P2X7R expression in the eye. We hope to demonstrate therapeutic efficacy for our novel P2X7R antagonists in treating eye disorders such as age-related macular degeneration.

#### Annette Nicke, Walther Straub Institute of Pharmacology and Toxicology, Ludwig-Maximilians-Universität Munich, Munich, Germany

We are interested in cell-type specific localisation of P2X7Rs and molecular mechanisms of its activation and signalling. We have various mutants, chimeras, and (fluorescently) tagged versions of human mouse and rat P2X7Rs for heterologous expression in *Xenopus laevis* oocytes and mammalian cells as well as a stably transfected P2X7R-EGFP HEK cell line and floxed P2X7R, P2X7R knockout, and BAC-transgenic P2X7R-EGFP reporter mouse models. We use protein biochemistry (BN-PAGE, cross-linking) in combination with mass spectrometry, functional/biophysical approaches (two-electrode voltage-clamp, voltage clamp fluorometry), and confocal microscopy. Together with collaborators within PRESTO, we aim to validate P2X7R as a drug target.

#### Christa E. Müller, Pharmaceutical Institute, Department of Pharmaceutical & Medicinal Chemistry, and PharmaCenter Bonn, University of Bonn, Germany

Our research is focused on medicinal chemistry and in vitro pharmacology of membrane proteins involved in purinergic signalling (mainly purine receptors and ectonucleotidases). Moreover, we have recently embarked on structural biology projects to elucidate binding interactions of compounds with their protein targets. We also perform quantitative analysis to determine drug levels in organs and tissues, e.g. of mice and rats. In the P2XR field, we established calcium influx assays for all P2XR subtypes of different species. Our main focus is on the P2X4R for which we have developed highly potent and selective tool compounds, including a radioligand, and drugs. We have also been developing potent P2X1R and P2X2R antagonists, and we are looking forward to collaborations with biologists and pharmacologists within the PRESTO research family.

#### Diego Dal Ben, School of Pharmacy, Medicinal Chemistry Unit, University of Camerino, Italy

Our research focuses on the rational design and synthesis of potentially novel P2XR ligands to be used as pharmacological tools, probes, and drugs. In previous years, we have already developed several compounds able to modulate purinergic receptor function. We have new molecular modelling and chemistry laboratories fully equipped with drug design software and all the required tools for chemical synthesis and characterisation of compounds. We hope to form new collaborations with PRESTO participants and we plan to share our expertise and studies with all the participants of the Action, to improve the research on P2XRs as therapeutic targets.

#### Vanessa D’Antongiovanni, Department of Clinical and Experimental Medicine, University of Pisa, Italy

Our research is focused on the pathophysiological mechanisms underlying intestinal disorders occurring in the presence of digestive and extra-digestive diseases (i.e. chronic intestinal inflammation, obesity, and neurodegenerative diseases). In this context, the research is focused on the role of the enteric bacteria-neuro-immune network. We have several human and murine cell lines (intestinal epithelial cells, macrophages, enteric glia) and in vivo models of inflammatory bowel diseases, obesity, and neurodegenerative disorders. We have studied the role of P2X4R in the pathophysiological mechanisms underlying the intestinal inflammation associated with colitis using several techniques: ELISA, western blots, co-culture, and immunofluorescence. During PRESTO, we would like to create new collaborations and share skills and expertise with PRESTO members.

#### Eric Boué-Grabot, Institute for neurodegenerative diseases (IMN), CNRS and University of Bordeaux, France

Our research focuses on the regulation and function of P2XRs in the healthy and diseased brain. We studied the properties and function of P2XRs by electrophysiology and imaging in a recombinant system (*Xenopus* oocytes) and in neurons/brain slices. We have numerous (WT, tagged, mutated) P2XR constructs and have developed several conditional transgenic knock-in and knock-out P2X4R mice to understand how cell-specific P2X4Rs contribute to several brain disorders including neurodegenerative diseases, chronic pain, or anxiety. We hope through new collaborations we will help PRESTO members to define cell-specific P2X4Rs as a target in other peripheral diseases. We also hope that knowledge exchange will advance the development of tools/modulators to target P2X4R function and/or surface trafficking for patient benefit.

#### Petros P. Petrou, Biochemical Genetics Department, The Cyprus Institute of Neurology & Genetics, Cyprus

Our research explores novel aspects of glycogen metabolism in physiological and pathological conditions. We employ cell culture and mouse models and apply histological, molecular, and biochemical techniques. We have recently established a link between endoplasmic reticulum (ER) stress and glycogen metabolism and intracellular clustering in a cell model of skeletal muscle differentiation. Given that the implication of P2XRs in glycogen metabolism and ER stress is underexplored, we aim to study P2XR signalling in this process. Through PRESTO, we envisage to exchange knowledge and experimental tools and establish collaborations to shed light into novel aspects of P2XR function.

#### Victoria Maneu, Neurobiology of the Visual System Laboratory, University of Alicante

We study retinal degeneration in several models. We analyse factors involved in degeneration and search for therapies that reverse or slow its progression. We perform functional and anatomical analysis of the retina (electroretinograms, optomotor test, immunohistochemical analysis) and apply molecular and cellular biology techniques for the study of mediators of inflammation and oxidative stress (RT-PCR, Western blot, flow cytometry). We have detected increased expression of P2XRs in our models of retinal dystrophies and try to use P2XR antagonists as a possible therapy. We expect to contact with other groups, learn, and contribute with our work and understanding in this field.
